# Expression Pattern of Dab1, Reelin, PGP9.5 and Sox2 in the Stomach of *Yotari* (*Dab1^−/−^*) Mice

**DOI:** 10.3390/genes16091013

**Published:** 2025-08-27

**Authors:** Petar Todorović, Nela Kelam, Anita Racetin, Natalija Filipović, Yu Katsuyama, Mirna Saraga-Babić, Katarina Vukojević

**Affiliations:** 1 Department of Anatomy, Histology and Embryology, School of Medicine, University of Split, 21000 Split, Croatia; petar.todorovic@mefst.hr (P.T.); nela.kelam@mefst.hr (N.K.); anita.racetin@mefst.hr (A.R.); natalija.filipovic@mefst.hr (N.F.); katarina.vukojevic@mefst.hr (K.V.); 2 Department of Anatomy, Shiga University of Medical Science, Otsu 520-2192, Japan; kats@belle.shiga-med.ac.jp; 3 Department of Anatomy, School of Medicine, University of Mostar, 88000 Mostar, Bosnia and Herzegovina; 4 Mediterranean Institute for Life Sciences, University of Split, 21000 Split, Croatia; 5 Center for Translational Research in Biomedicine, School of Medicine, University of Split, 21000 Split, Croatia

**Keywords:** Reelin–Dab1 signaling, *yotari*, gastric development, Sox2, PGP9.5, epithelial–mesenchymal interaction

## Abstract

**Background/Objectives**: The Reelin–Dab1 signaling pathway, known for its crucial role in neurodevelopment, particularly in neuronal migration and the formation of cortical layers, has been a subject of extensive research. However, its involvement in gastrointestinal organogenesis is a relatively unexplored area. Our study investigates the expression patterns of Dab1, Reelin, PGP9.5, and Sox2 during stomach development in *yotari* (*Dab1^−/−^*) mice and aims to shed light on how *Dab1* inactivation affects epithelial–mesenchymal signaling dynamics, thereby contributing to a deeper understanding of this pathway’s non-neural functions. **Methods**: Embryonic stomach tissues from *yotari* and wild-type mice, collected at developmental stages E13.5 and E15.5, were examined by immunofluorescenceto evaluate the difference in expression of Dab1, Reelin, PGP9.5, and Sox2. Semi-quantitative scoring and quantitative image analysis were used to assess protein localization and intensity within epithelial and mesenchymal compartments. **Results**: Dab1 expression was significantly increased in both the epithelium and mesenchyme of *yotari* mice at E13.5 and E15.5. Reelin expression in the epithelium showed a visible but statistically non-significant decrease in *yotari* at E15.5, while mesenchymal expression remained low and significantly lower than controls. PGP9.5 expression was significantly reduced in *yotari* epithelium at E13.5, then strongly upregulated at E15.5. Mesenchymal PGP9.5 remained consistently high. Sox2 showed no statistically significant changes but increased semi-quantitatively in *yotari* epithelium and mesenchyme at E15.5. These findings highlight compartment-specific disruptions and potential compensatory mechanisms following *Dab1* inactivation. **Conclusions**: Our findings indicate that *Dab1* deficiency leads to distinct molecular changes in epithelial and mesenchymal compartments of the developing stomach. The Reelin–Dab1 axis appears critical for epithelial–mesenchymal coordination, while PGP9.5 and Sox2 upregulation in *yotari* mice may represent potential compensatory responses that could support epithelial integrity, although this remains speculative without functional validation.

## 1. Introduction

The enteric nervous system (ENS), often called the “second brain,” is a complex network of neurons and glial cells that controls gastrointestinal functions such as motility, secretion, and blood flow. Originating from neural crest cells, the ENS develops through migration, proliferation, and differentiation [1,2]. Though semi-autonomous, it communicates bidirectionally with the central nervous system to regulate gut homeostasis [1]. Recent studies have also demonstrated that the ENS regulates gastrointestinal physiology, immune responses and contributes to neurological disease through its bidirectional communication with the central nervous system in the gut–brain axis [3,4].

The cytoplasmic adaptor protein Disabled-1 (Dab1) plays a central role in the Reelin signaling pathway. Dab1 is phosphorylated in response to Reelin binding, initiating downstream signaling essential for neuronal migration and cortical layer formation [5,6]. *Yotari (Dab1^−/−^)* mice display a phenotype similar to *Reelin* knockouts, with tremors, motor instability, and early lethality [7,8]. Although Dab1’s role in neural development is well established, its function in gastrointestinal or stomach development has not been investigated. However, studies demonstrated the involvement of DAB1 in mesenchymal–epithelial interactions during kidney organogenesis, where DAB1 modulates epithelial differentiation [9].

Reelin is an essential glycoprotein located outside the cell that plays central role in brain development by guiding neuron movement, promoting axon elongation, and supporting the formation of synapses [10]. It signals through ApoER2 and VLDLR receptors to activate Dab1, orchestrating cytoskeletal dynamics necessary for neuronal placement [11]. Beyond neurodevelopment, it is expressed in multiple neuronal subtypes, including glutamatergic and GABAergic cells in the cortex, hippocampus, and cerebellum [12]. Reelin was found to be expressed during embryonic development and has also been detected in the stomach epithelium of mice [13].

PGP9.5 (ubiquitin C-terminal hydrolase L1, UCH-L1) is a neuronal enzyme of the ubiquitin–proteasome system that maintains protein homeostasis by promoting the clearance of ubiquitinated proteins, thereby preserving synaptic integrity and neuronal function [14]. It is a well-established pan-neuronal marker in both developing and adult gastrointestinal tissues, labeling enteric neurons and migrating neural-crest-derived precursors [15,16]. In the human colon, Reelin, its receptors (VLDLR and ApoER2), and the adaptor protein Dab1 are present in the tunica muscularis and myenteric ganglia [17,18]. Dual-label immunohistochemistry has shown co-localization of Reelin with PGP9.5 in neuronal somata and processes of myenteric ganglia and intramuscular nerve fibers, confirming its specific neuronal localization [18]. This anatomical association directly links Reelin expression to the neuronal network of the enteric nervous system (ENS) and supports the relevance of PGP9.5 assessment in studies investigating the Reelin–Dab1 signaling pathway during ENS development [18].

SOX2 is considered one of the crucial transcription factors responsible for pluripotency and differentiation in stem cells [19]. Sox2 maintains the pluripotent state of ENS progenitor cells and contributes to their differentiation into neuronal and glial lineages [20]. Its expression has been noted in neural and epithelial tissues, among many others, which is essential for maintaining and repairing these tissues [21]. Sox2 marks self-renewing epithelial stem cells in the stomach and supports glandular development [22]. Reelin–Dab1 signaling, although better known for its neural functions, also regulates epithelial proliferation, migration, differentiation, and apoptosis in the intestinal crypt–villus unit [23]. Therefore, measuring SOX2 expression may uncover changes in gastric epithelial progenitor activity or compensatory mechanisms following Dab1 disruption.

This study investigates the expression patterns of Dab1, Reelin, PGP9.5 and Sox2 in the embryonic stomach of *yotari* (*Dab1^−/−^*) mice at E13.5 and E15.5. The timepoints E13.5 and E15.5 were selected because they represent critical phases of gastric morphogenesis, including epithelial remodeling, mesenchymal compartment specialization, and the differentiation of neural crest–derived enteric neurons. Earlier stages such as E11–E12 correspond to a rudimentary tubular gut with minimal compartmentalization and incomplete colonization by enteric progenitors, making compartment-specific quantitative analysis is technically challenging and less informative for our study objectives [24].

The *yotari* mutation arises from the replacement of two complete exons and part of another exon in the *Dab1* gene with a long interspersed nuclear element (L1) fragment. This alteration produces a mutant *Dab1* mRNA that fails to translate into functional DAB1 protein [25]. Although Dab1 protein fragments may be present, they are not phosphorylated and lack normal function, making *yotari* mice functional null mutants for *Dab1* rather than classical knockouts [7].

Through analysis of epithelial and mesenchymal layers, we aim to better understand the developmental roles and uncover compensatory mechanisms activated due to functional silencing of *Dab1*. Because transcriptional regulation plays a critical role in coordinating gastric development, we also consider potential upstream transcription factors and regulatory mechanisms that could contribute to the altered expression patterns observed in *yotari* mice.

## 2. Materials and Methods

### 2.1. Ethical Approval

The use of animals in this research was approved by the Shiga University of Medical Science’s Guidelines for the Care and Use of Laboratory Animals. The study was conducted in accordance with the principles of the Declaration of Helsinki and approved by the Ethical Committee of the University of Split School of Medicine (Class: 003-08/23-03/0015; Protocol Code No.: 2181-198-03-04-23-0073; Date of Approval: 27 September 2023).

### 2.2. Sample Collection

Homozygous *yotari* (*Dab1^−/−^*) mice, characterized by an autosomal recessive mutation in the *Dab1* gene, were used in this study. These animals typically display tremors, impaired motor coordination, and early postnatal mortality [7]. Both *yotari* and C57BL/6N control (ctrl) mice were maintained under standard laboratory conditions in individual polycarbonate cages, with food and water available ad libitum. The housing environment was temperature-controlled at approximately 23 °C, with minor fluctuations of up to ±2 °C, and maintained under a 12 h light/12 h dark cycle. The primer sequences used for genotyping are outlined below:

*yotari*—GCCCTTCAGCATCACCATGCT and CAGTGAGTACATATTGTGTGAGTTCC;

control specimens—GCCCTTCAGCATCACCATGCT and CCTTGTTTCTTTGCTTTAAGGCTGT.

The gravid mice were sacrificed on embryonic days 13.5 (E13.5) and 15.5 (E15.5) to obtain their embryos. We analyzed three mice from each genotype (*yotari* and control specimens) for each designated timepoint. These were three individual embryos per genotype per developmental stage (E13.5 and E15.5), yielding a total of 12 biological replicates (*n* = 3 per group per stage). The sample size was determined a priori using Mead’s resource equation, a method widely applied in laboratory animal research to ensure adequate statistical power while adhering to ethical principles of reduction. Based on two developmental stages and two genotypes, the equation recommended three embryos per group, producing an error degree of freedom (E) within the acceptable range of 10–20, in line with ARRIVE guidelines. Initially, the animals were deeply anesthetized using pentobarbital. They were then transcardially perfused with phosphate-buffered saline (PBS, pH 7.2), followed by 4% paraformaldehyde (PFA) in 0.1 M PBS. Embryos were fixed separately in 4% PFA in 0.1 M PBS overnight.

### 2.3. Immunofluorescence Staining

After fixation, the tissue underwent a dehydration process through a graded ethanol series. The dehydrated samples were embedded in paraffin, and thin slices approximately 5 µm thick were prepared and mounted onto microscope slides. To check tissue preservation, every tenth section was stained with hematoxylin and eosin. Before immunostaining, paraffin was removed from the sections using xylene, followed by rehydration in decreasing concentrations of ethanol. Antigen retrieval was carried out by heating the slides in 0.01 M citrate buffer (pH 6.0) at 95 °C for 30 min in a water steamer, then cooling them slowly to room temperature. After rinsing with 0.1 M PBS, a commercial protein-blocking reagent (ab64226, Abcam, Cambridge, UK) was applied for 20 min to minimize nonspecific binding. Primary antibodies (listed in Table 1) were applied to the sections and incubated overnight in a humidity chamber. On the following day, the slides were washed with PBS and exposed for 1 h to the corresponding secondary antibodies (Table 1). After additional PBS washes, the nuclei were counterstained with 4′,6-diamidino-2-phenylindole (DAPI), and the preparations were mounted with Immu-Mount (Thermo Shandon, Pittsburgh, PA, USA).

A preadsorption test was performed to confirm the specificity of the antibodies. Each primary antibody was preincubated with an excess of its corresponding peptide antigen in a blocking solution at a precisely determined concentration before application to the sections. The absence of any immunofluorescent signal confirmed the specificity of the antibodies. Furthermore, when primary antibodies were omitted from the immunofluorescence protocol, there was no evidence of nonspecific binding by the secondary antibodies or any false-positive results. The anti-Dab1 antibody used in this study (ab78200, Abcam, Cambridge, UK) is a phospho-specific polyclonal antibody raised against a synthetic peptide corresponding to the region surrounding phosphorylated tyrosine 232 (pY232) of human DAB1 (Table 1). This epitope is conserved in the mouse sequence and preserved in *yotari* mice despite the *Dab1* mutation. Preadsorption controls were performed by incubating the antibody with a 10-fold molar excess of its immunizing phosphopeptide before application. This completely abolished all immunofluorescent signal in both control and *yotari* stomach sections, confirming sequence-specific binding. Additional controls omitting the primary antibody yielded no detectable background fluorescence. Moreover, the Dab1 staining pattern observed in control tissues matched previously reported localization in mouse embryonic organs, including the gastrointestinal tract [9,23], further supporting antibody specificity.

### 2.4. Data Acquisition and Analysis

Hematoxylin–Eosin (H&E) stained images of stomach tissues from *yotari* and control specimens at E13.5 and E15.5 were obtained to assess potential morphological differences. The slides were examined under a light microscope (BX40, Olympus, Tokyo, Japan) fitted with a camera (DP27, Olympus, Tokyo, Japan).

The stomach sections stained by immunofluorescence were analyzed using an epifluorescence microscope (BX51, Olympus, Tokyo, Japan) equipped with a Nikon DS-Ri2 camera (Nikon Corporation, Tokyo, Japan) and NIS-Elements F software (version 5.22.00). For protein immunoexpression quantification, at least 10 non-overlapping visual fields were imaged at 40× magnification using constant exposure settings. Images were subsequently processed with ImageJ software (version 1.53o; National Institutes of Health, Bethesda, MD, USA) and Adobe Photoshop (version 21.0.2; Adobe, San Jose, CA, USA).

Initially, Adobe Photoshop was employed to remove the background signal using the “Levels” function. Subsequently, the epithelium was selected with the Lasso tool and isolated from the mesenchyme by cutting it from the original image and placing it into a blank canvas of the exact dimensions.

The images were analyzed as we described previously [26,27,28]. The separated images were loaded into ImageJ, duplicated, and divided into their respective color channels. To enhance the purity of the green signal, the red channel was subtracted.

The images were then duplicated, and a median filter (radius 10) was applied to one copy for all antibodies. Filtered images were subtracted from their unfiltered counterparts to isolate the positive signal. The resulting images were converted to 8-bit format and thresholded using the “triangle” method, after which the area percentage was calculated with the “Analyze Particles” function.

Upon examination of the analyzed images, it was discovered that certain regions lacked the presence of tissue, resulting in an area percentage measurement that was lower than the actual area percentage. To rectify this issue, the Magic Wand tool in Adobe Photoshop was utilized to determine the total number of pixels (px) present in the images and the number of empty space pixels. Subsequently, the corrected area percentage was calculated as described previously [29] and used for statistical analyses.

This image analysis protocol, including triangle thresholding and empty-space correction, has been validated and applied in multiple previous peer-reviewed studies from our group [27,30,31]. Quantitative results obtained using this approach have shown excellent agreement with independent visual scoring by experienced histologists.

### 2.5. Statistical Analysis and Semi-Quantitative Scoring Rationale

All statistical analyses were performed using GraphPad Prism 9.0.0 (GraphPad Software, San Diego, CA, USA). Protein expression was quantified as the percentage of positive pixels within epithelial or mesenchymal compartments, measured from thresholded immunofluorescence images. Differences between groups were assessed using a two-way analysis of variance (ANOVA) with genotype (yotari vs. control) and developmental stage (E13.5 vs. E15.5) as independent variables. This test was selected because it evaluates not only the main effect of each factor separately, but also whether there is an interaction between them. When a significant main effect or interaction was detected, Tukey’s multiple comparison post hoc test was applied to determine which group pairs differed while controlling for the family-wise error rate. Data are expressed as mean ± standard deviation (SD), and *p* < 0.05 was considered statistically significant.

In addition to objective image-based quantification, a semi-quantitative scoring system was used to capture overall staining intensity patterns and localization features that may not be fully reflected in pixel counts. The scoring scale was defined as: no reactivity (–), mild (+), moderate (++), and strong (+++). Inter-observer agreement for the semi-quantitative scores was further evaluated in detail using the intraclass correlation coefficient (ICC) with a two-way random effects model, absolute agreement definition (ICC [1,2]) [32], as recommended for reliability testing in quantitative research [33]. Three independent observers, blinded to genotype and developmental stage, scored the same set of 48 images (4 proteins × 2 compartments × 2 stages × 3 biological replicates). ICC values and 95% confidence intervals (CIs) were calculated in GraphPad Prism v9.0.0. The overall ICC was 0.83 (95% CI: 0.79–0.87), with per-protein ICC values ranging from 0.80 to 0.86, indicating excellent agreement according to Cicchetti’s criteria [33].

## 3. Results

The protein expression patterns of Dab1, Reelin, PGP9.5, and Sox2 were evaluated in the embryonic stomach tissue of *yotari* (*Dab1^−/−^*) mice and control specimens at developmental days E13.5 and E15.5. Immunohistochemical staining and quantification of positive area percentage were analyzed separately for the epithelium and mesenchyme.

### 3.1. Preserved Gastric Histology in Wild-Type and Yotari Embryonic Stomachs

**Figure 1:**
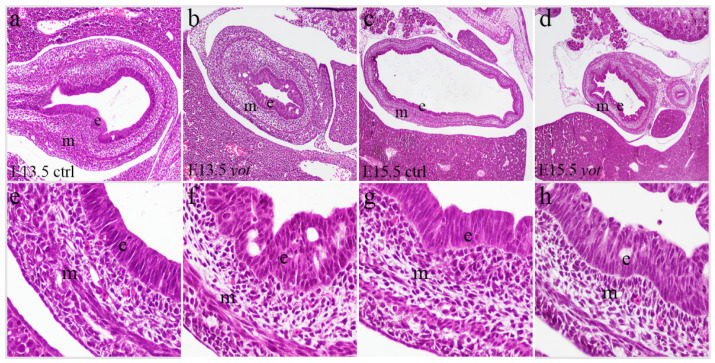
Hematoxylin-eosin (H&E) staining of the control specimens (ctrl) and *yotari* (*yot*) stomach. H&E staining of the ctrl (**a**,**e**) and *yot* (**b**,**f**) stomach at embryonic day 13.5 (E13.5). H&E staining of the ctrl (**c**,**g**) and *yot* (**d**,**h**) stomach at embryonic day 15.5 (E15.5). Images were taken at an objective magnification of ×4 (**c**,**d**), ×20 (**a**,**b**), and ×40 (**e**–**h**). e = epithelium, m = mesenchyme.

Hematoxylin-eosin (H&E) stained sections revealed no significant morphological differences in overall stomach structure between control specimens and *yotari* mice (Figure 1). The mucosal, submucosal, muscular, and serosal layers were well-preserved in all specimens, with no detectable histological abnormalities or disruptions in tissue integrity between the examined phenotypes. This evaluation was qualitative only; no quantitative morphometric analysis was performed, as morphological assessment was not a primary objective of this study.

### 3.2. Dab1 Is Significantly Upregulated in Yotari Mice During Gastric Development

**Figure 2:**
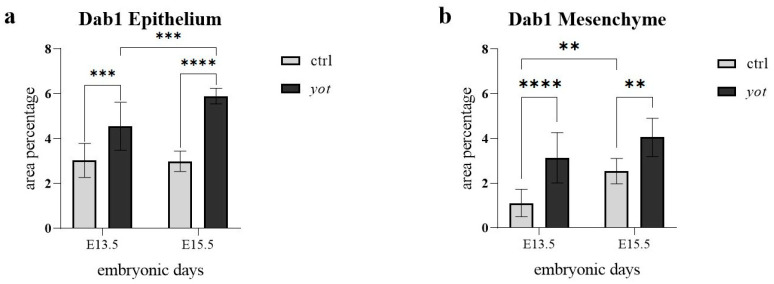
The area percentage of Dab1 in control specimens (ctrl) and *yotari* mice (*yot)* per structure (epithelium and mesenchyme) throughout different stages of developing stomach. Results are presented as mean ± standard deviation (SD), based on three biological replicates per group (*n* = 3). Statistical analysis was performed using two-way ANOVA followed by Tukey’s multiple comparison test. The following symbols indicate levels of statistical significance: ** *p* < 0.01; *** *p* < 0.0001; **** *p* < 0.00001. Exact mean differences, 95% confidence intervals, and adjusted *p*-values for all pairwise comparisons are provided in Supplementary Tables S1 and S2.

In the epithelium (Figure 2a), *yotari* mice at E13.5 showed significantly higher Dab1 expression than control mice at the same stage (*** *p* < 0.0001), with semi-quantitative scoring of ++ for control and +++ for *yotari* (Table 2). A significant increase was also observed within the *yotari* group from E13.5 to E15.5 (**** *p* < 0.00001), although both timepoints were scored +++, indicating an increase in quantitative intensity. At E15.5, *yotari* embryos showed significantly higher Dab1 expression than controls (*** *p* < 0.0001); both were scored +++, but *yotari* had a higher signal (Figure 2a, Table 2). There was no statistically significant difference between control E13.5 and control E15.5, despite the semi-quantitative increase in protein reactivity from moderate to strong.

In the mesenchyme (Figure 2b), Dab1 expression in *yotari* mice at E13.5 was significantly higher than in controls (**** *p* < 0.00001), reflected by a shift in scoring from ++ in control to +++ in *yotari*. In control mice, Dab1 significantly increased from E13.5 to E15.5 (** *p* < 0.01), with scores also rising from ++ to +++ (Figure 2b, Table 2). At E15.5, *yotari* mice exhibited significantly higher mesenchymal Dab1 expression than controls (** *p* < 0.01), even though both were scored +++. No statistically significant difference was found between *yotari* E13.5 and *yotari* E15.5, with both timepoints consistently scored as +++ (Figure 2b, Table 2).

**Figure 3:**
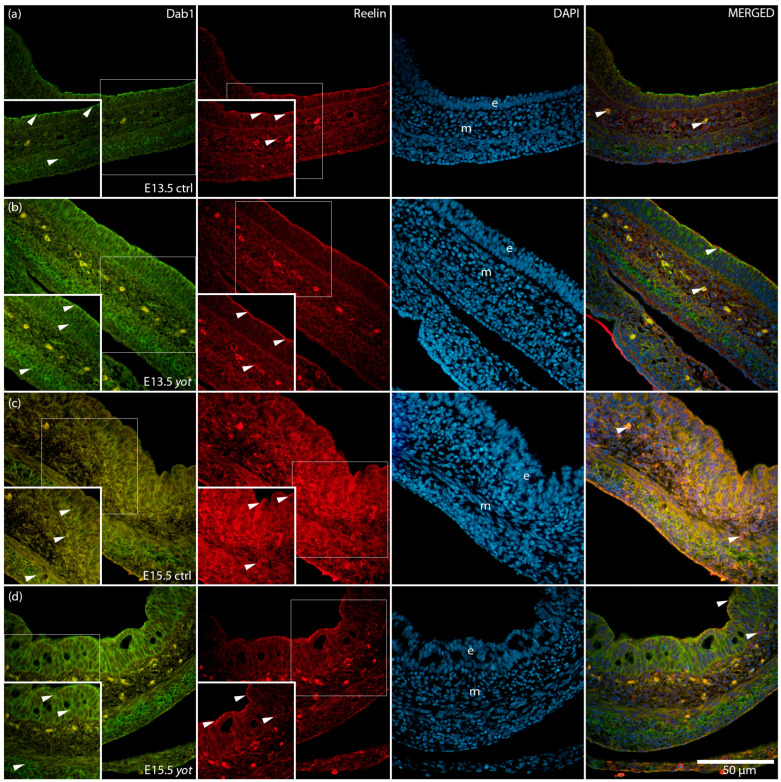
Immunofluorescence staining of Disabled 1 (Dab1) and Reelin, merged with 4′,6-diamidino-2-phenylindole (DAPI), in developing control (ctrl) and *yotari* (*yot*) stomach tissue (**a**–**d**). Comparative expression of Dab1 and Reelin in the stomach at embryonic day 13.5 (E13.5) and 15.5 (E15.5) is shown (**a**–**d**). Positive staining of Dab1 and Reelin is indicated by arrows in each substructure of the stomach. Merged images show co-localization of the signals (arrows). Epithelium (e) and mesenchyme (m) are labeled at both timepoints (**a**–**d**). Representative images were selected from three biological replicates per group (*n* = 3). Magnification: ×40; scale bar: 50 µm.

The spatial distribution and co-localization of Dab1 and Reelin were visualized using immunofluorescence, confirming their differential localization across the epithelium and mesenchyme (Figure 3).

### 3.3. Reelin Expression Is Maintained in Epithelium but Significantly Reduced in Mesenchyme of Yotari Mice

**Figure 4:**
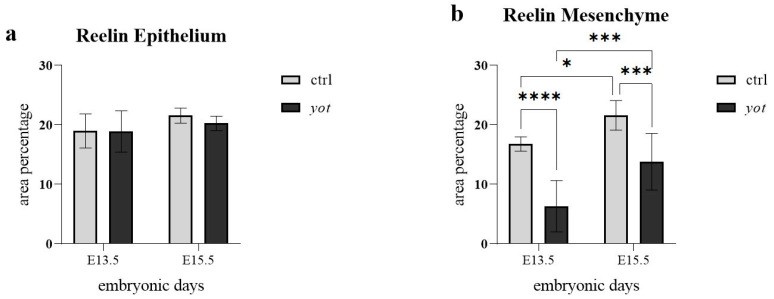
The area percentage of Reelin in control specimens (ctrl) and *yotari* mice (*yot)* per structure (epithelium and mesenchyme) throughout different stages of developing stomach. Results are presented as mean ± standard deviation (SD), based on three biological replicates per group (*n* = 3). Statistical analysis was performed using two-way ANOVA followed by Tukey’s multiple comparison test. The following symbols indicate levels of statistical significance: * *p* < 0.05; *** *p* < 0.0001; **** *p* < 0.00001. Exact mean differences, 95% confidence intervals, and adjusted *p*-values for all pairwise comparisons are provided in Supplementary Tables S1 and S2.

In the epithelium there were no statistically significant differences in Reelin expression between control and *yotari* mice at either timepoint (Figure 4a). Specifically, expression levels were comparable between control and *yotari* at E13.5, as well as between control and *yotari* at E15.5. Additionally, no significant differences were observed within the *yotari* group between E13.5 and E15.5, nor between control E15.5 and *yotari* E15.5 (Figure 4a). This is in line with semi-quantitative scoring, which showed moderate (++) expression in both groups at E13.5. At E15.5, control remained at ++, while *yotari* decreased to mild (+), indicating a drop in intensity that was visible but not statistically significant (Table 2).

In the mesenchyme (Figure 4b), several statistically significant differences were observed. At E13.5, control mice exhibited significantly higher Reelin expression than *yotari* (**** *p* < 0.00001), although both were scored mild (+) (Table 2). Between stages in the control group, Reelin expression increased significantly from E13.5 to E15.5 (* *p* < 0.05), with semi-quantitative scores rising from + to ++ (Figure 4b, Table 2). At E15.5, control mice also had significantly higher Reelin expression than *yotari* (*** *p* < 0.0001), with scores of ++ in control and + in *yotari*. Within the *yotari* group, Reelin expression increased significantly from E13.5 to E15.5 (*** *p* < 0.0001), though both timepoints were scored mild (+), indicating a measurable quantitative increase despite unchanged categorical intensity (Figure 4b, Table 2).

### 3.4. PGP9.5 Expression Is Reduced in the Epithelium and Mesenchyme of Yotari Embryos During Early Gastric Development

**Figure 5:**
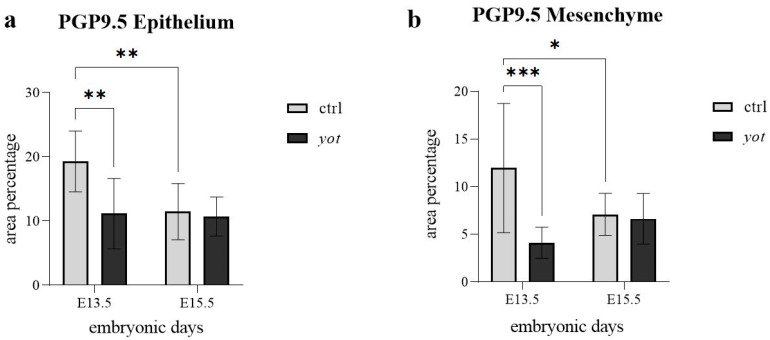
The area percentage of PGP 9.5 in control specimens (ctrl) and *yotari* mice (*yot)* per structure (epithelium and mesenchyme) throughout different stages of developing stomach. Results are presented as mean ± standard deviation (SD), based on three biological replicates per group (*n* = 3). Statistical analysis was performed using two-way ANOVA followed by Tukey’s multiple comparison test. The following symbols indicate levels of statistical significance: * *p* < 0.05; ** *p* < 0.01; *** *p* < 0.0001. Exact mean differences, 95% confidence intervals, and adjusted *p*-values for all pairwise comparisons are provided in Supplementary Tables S1 and S2.

In the epithelium (Figure 5a), control mice at E13.5 showed significantly higher PGP9.5 expression than *yotari* (** *p* < 0.01), in agreement with semi-quantitative scores of ++ in control and + in *yotari* (Table 2). Within the control group, epithelial PGP9.5 expression significantly decreased from E13.5 to E15.5 (** *p* < 0.01), with corresponding scores dropping from ++ to +. No statistically significant difference was observed within the *yotari* group between E13.5 and E15.5, despite the semi-quantitative increase from + to +++ (Figure 5a, Table 2). Additionally, there was no significant difference between control and *yotari* mice at E15.5, even though the scoring differed (+ in control, +++ in *yotari*), suggesting high variability in the *yotari* group (Figure 5a, Table 2).

In the mesenchyme (Figure 5b), control embryos at E13.5 displayed significantly higher expression than *yotari* (*** *p* < 0.0001), though both were scored strong (+++), indicating a quantitative but not categorical difference (Table 2). Within the control group, PGP9.5 expression decreased slightly from E13.5 to E15.5 (* *p* < 0.05), though the semi-quantitative score remained +++ at both timepoints. No statistically significant differences were found between control and *yotari* at E15.5, or within the *yotari* group across timepoints, which were also scored +++ throughout (Figure 5b, Table 2).

**Figure 6:**
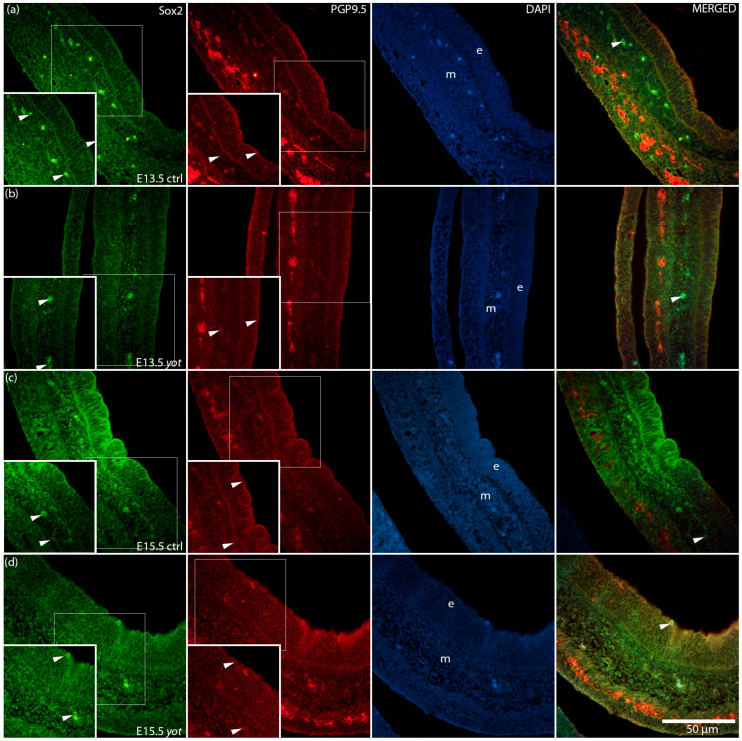
Immunofluorescence staining of SRY-box transcription factor 2 (Sox2) and Protein Gene Product 9.5 (PGP9.5), merged with 4′,6-diamidino-2-phenylindole (DAPI), in developing control (ctrl) and *yotari* (*yot*) stomach tissue (**a**–**d**). Comparative expression of Sox2 and PGP9.5 in the stomach at embryonic day 13.5 (E13.5) and 15.5 (E15.5) is shown (**a**–**d**). Positive staining of Sox2 and PGP9.5 is indicated by arrows in each substructure of the stomach. Merged images show co-localization of the signals (arrows). Representative images were selected from three biological replicates per group (*n* = 3). Epithelium (e) and mesenchyme (m) are labeled at both timepoints (**a**–**d**). Magnification: ×40; scale bar: 50 µm.

To further investigate epithelial differentiation and neural marker distribution, we examined the expression and co-localization of Sox2 and PGP9.5 in control and *yotari* stomachs at E13.5 and E15.5 (Figure 6). In control embryos, Sox2 was predominantly localized to the epithelial layer, while PGP9.5 was detected in both the epithelium and underlying mesenchyme. In *yotari* samples, an increased overlap of Sox2 and PGP9.5 signals was observed within the epithelium.

### 3.5. Sox2 Expression Patterns in the Embryonic Stomach Show No Significant Genotype- or Stage-Dependent Differences

**Figure 7:**
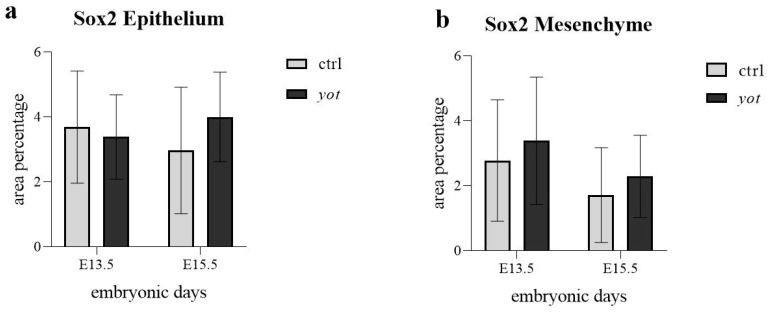
The area percentage of Sox2 in control specimens (ctrl) and *yotari* mice (*yot)* per structure (epithelium and mesenchyme) throughout different stages of developing stomach. Results are presented as mean ± standard deviation (SD), based on three biological replicates per group (*n* = 3). Statistical analysis was performed using two-way ANOVA followed by Tukey’s multiple comparison test. Exact mean differences, 95% confidence intervals, and adjusted *p*-values for all pairwise comparisons are provided in Supplementary Tables S1 and S2.

In the epithelium (Figure 7a), no statistically significant differences in Sox2 expression were observed between any of the compared groups. Expression levels were not significantly different between control and *yotari* at E13.5, between control embryos from E13.5 to E15.5, or between *yotari* embryos across the same developmental interval. Likewise, no significant difference was detected between control and *yotari* at E15.5. Despite these findings, semi-quantitative analysis showed variability: control mice exhibited a decrease from moderate (++) at E13.5 to mild (+) at E15.5, while *yotari* mice showed an increase from mild (+) to strong (+++) (Table 2). These shifts reflect observable trends in signal intensity, though they were not statistically significant.

In the mesenchyme (Figure 7b), Sox2 expression similarly showed no statistically significant differences between any comparison groups, including control versus *yotari* at E13.5, within controls or *yotari* across time, and between genotypes at E15.5. Semi-quantitative scoring indicated that control mice decreased from moderate (++) at E13.5 to mild (+) at E15.5, while *yotari* mice increased from mild (+) to strong (+++) over the same period (Figure 7b, Table 2). These shifts were evident visually but did not reach statistical significance.

### 3.6. Summary of Semi-Quantitative Expression Patterns

A semi-quantitative overview of protein expression across developmental stages and genotypes is presented in Table 2. Dab1 showed a consistent increase in both epithelium and mesenchyme of *yotari* mice compared to controls at E13.5, with strong (+++) reactivity maintained at E15.5 in both groups. Reelin expression remained moderate (++) in the epithelium of controls across both stages but decreased to mild (+) in *yotari* at E15.5. In the mesenchyme, Reelin remained mild (+) in *yotari* at both timepoints, while it increased from mild (+) to moderate (++) in controls. PGP9.5 was reduced in the epithelium of *yotari* at E13.5 compared to controls, but showed a marked increase to strong (+++) by E15.5, in contrast to controls, which decreased over time. In the mesenchyme, PGP9.5 expression remained consistently strong (+++) in both genotypes and timepoints. Sox2 expression in both epithelium and mesenchyme was reduced in *yotari* at E13.5 compared to controls, but increased to strong (+++) by E15.5, while in controls it declined over time. The overall inter-observer reliability was excellent, with a pooled ICC of 0.83 (95% CI: 0.79–0.87) across all proteins, compartments, and developmental stages. ICC values for individual proteins ranged from 0.80 to 0.86, confirming high scoring consistency between observers.

## 4. Discussion

The enteric nervous system plays a vital role in regulating gastrointestinal functions through a complex network of neurons and glial cells that develop from neural crest-derived progenitors. Molecular factors such as Dab1, Reelin, PGP9.5, and Sox2 are key regulators of ENS development, influencing processes like neuronal migration, differentiation, and progenitor cell maintenance. To our knowledge, this study is among the first to investigate the expression and role of Dab1 and its associated proteins, including Reelin, PGP9.5, and Sox2, in the developing stomach of the *yotari* mice. By filling this gap in the literature, we provide novel insights into how altered Reelin-Dab1 signaling may influence enteric nervous system formation and gastric tissue organization during embryogenesis. The protein expression of Dab1, Reelin, PGP9.5, and Sox2 was analyzed semi-quantitatively and quantitatively in *yotari* mice and control specimens.

Dab1 expression was significantly altered in the epithelium and the mesenchyme between observed phenotypes at both observed developmental stages. In the epithelium, *yotari* mice showed higher Dab1 expression than controls at E13.5 and E15.5, and expression increased significantly between E13.5 and E15.5 within *yotari*. Since the *Dab1* mutation in *yotari* mice leads to the production of a truncated, nonfunctional protein [9], the persistent epithelial signal may reflect the accumulation of a truncated, likely inactive form of Dab1. This interpretation is consistent with the known *yotari* mutation. Reports of truncated Dab1 isoforms in non-neuronal tissues suggest a potential role, but the findings remain speculative without biochemical confirmation, such as Western blotting or mass spectrometry.

Although no studies have directly examined Dab1 protein expression in the developing stomach, related work in other organs provides context. Racetin et al. demonstrated that DAB1 participates in mesenchymal–epithelial interactions during kidney development, influencing epithelial polarity and organization [9]. Their findings support the idea that Dab1 can influence epithelial organization even outside the nervous system. Our observation of increased Dab1 in the gastric mesenchyme may similarly reflect an adaptive response aimed at preserving epithelial stability when canonical signaling is impaired.

Gao et al. proposed that *Dab1* acts as a critical organizer of brain architecture by regulating neuronal migration [5]. While their work emphasized gene-level regulation via alternative splicing, the present study reports increased Dab1 protein abundance in *yotari* mesenchyme without evidence of active downstream signaling. This observation may reflect a passive accumulation of non-functional or hypofunctional *Dab1* isoforms, which could hypothetically serve structural or signaling-independent roles, consistent with studies showing truncated isoforms and altered subcellular localization in non-neuronal contexts [34].

Bock et al. demonstrated that Reelin-induced tyrosine phosphorylation of Dab1 enables its interaction with the PI3K regulatory subunit p85, activating downstream signaling pathways that modulate cytoskeletal dynamics and membrane-associated signaling [8]. Given the central role of cytoskeletal remodeling during embryogenesis, it is conceivable that even truncated or non-functional Dab1 might still participate in scaffolding or spatial organization, although this remains speculative. Further supporting this, García-Miranda et al. identified a truncated Dab1 isoform at epithelial junctions in the rodent intestine, co-localizing with clathrin and NWASP, suggesting a role in vesicular transport or spatial organization at the epithelial–mesenchymal boundary [35]. Vázquez-Carretero et al. showed that Dab1 and Reelin regulate crypt-villus homeostasis, with mutations affecting proliferation, migration, and apoptosis [23]. Therefore, the upregulation observed in our gastric model may be interpreted as a potential adaptive response rather than definitive evidence of functional compensation. Serrano-Morales et al. further noted Dab1 at epithelial-stromal borders in the human colon [17], suggesting a conserved role in mesenchymal–epithelial regulation.

Our findings of elevated Dab1 protein in the gastric mesenchyme of *yotari* mice align with these patterns, though we cannot confirm whether this truncated protein retains functional capability. These results suggest possible compensatory mechanisms during gastric development in response to *Dab1* inactivation, although such interpretations remain speculative without direct experimental support.

In contrast to Dab1, Reelin expression showed no statistically significant changes in the epithelium across any genotype or developmental stage, but was significantly altered in the mesenchyme. At E15.5, mesenchymal Reelin was significantly higher in control mice than in *yotari*, and increased across development in both genotypes, though *yotari* remained lower at both stages. This reduction in *yotari* mesenchyme may indicate a disrupted feedback loop or signaling inefficiency resulting from *Dab1* deficiency, though the exact nature of this disturbance remains uncertain.

Although not directly studied in the stomach, Halvorson et al. provided compelling evidence for Reelin’s role in intestinal epithelial turnover, stem cell differentiation, and migration via both canonical (VLDLR, ApoER2) and non-canonical (α3β1 integrins, EphB2) pathways [36]. Subepithelial myofibroblasts secrete Reelin, which influences crypt cells and maintains the integrity of epithelial barriers. Reelin deficiency in *reeler* mice disrupted tight junctions, decreased goblet cells, and impaired stem cell renewal, changes that are not found in the *yotari* gastric tissue. The mesenchymal Reelin reduction we observed may reflect impaired epithelial support, potentially contributing to Dab1 upregulation in both compartments as a compensatory mechanism.

Hirota et al. emphasized Reelin’s importance in neuronal migration and cortical layer formation throughcytoskeletal regulation, mechanisms that may also influence epithelial adhesion, polarity, and cell positioning [37]. Our data suggest that epithelial Reelin may play a supportive rather than dynamic role at later stages, while mesenchymal signaling appears more actively regulated during stomach development.

Niu et al. demonstrated that Reelin promotes dendritic spine formation and postsynaptic protein localization in hippocampal neurons, supporting its role in stabilizing synaptic architecture [38]. These findings may extend to epithelial cells, where Reelin could influence cytoskeletal organization during development.

PGP9.5 expression was significantly higher in the epithelium of control mice at E13.5 than in *yotari*, and decreased significantly by E15.5 in control mice. In contrast, *yotari* epithelium showed no significant difference between E13.5 and E15.5. No differences were found between genotypes at E15.5. In the mesenchyme, control embryos had significantly higher PGP9.5 than *yotari* at E13.5, and expression declined by E15.5 in controls, while *yotari* remained stable. Semi-quantitative scores showed consistently strong staining intensity across all groups in the mesenchyme.

We note that at E13.5, the mesenchymal smooth muscle layer displayed strong PGP9.5 (UCHL1) immunoreactivity. As PGP9.5 is a classical pan-neuronal marker, its high signal in smooth muscle is unexpected. Although originally described as neuron-specific, previous reports have shown that PGP9.5 immunoreactivity can occasionally appear outside neuronal populations, particularly in smooth muscle, fibroblasts, and epithelial cells, either due to developmental expression or non-specific background depending on fixation and antibody conditions [15,39,40,41]. Furthermore, acute injury has been shown to induce PGP9.5 expression in non-neuronal epithelial cells, further underscoring that its appearance outside neuronal tissue may not always represent bona fide neuronal labeling [40]. Therefore, part of the strong mesenchymal labeling at E13.5 may reflect non-specific background rather than true neuronal expression. We interpret these findings with caution, while emphasizing that expected neuronal structures (e.g., submucosal and myenteric plexus regions) showed clear PGP9.5-positive fibers, supporting antibody functionality in our study. PGP9.5, a ubiquitin carboxyl-terminal hydrolase, is classically used as a neuronal marker involved in the ubiquitin-proteasome degradation system [14]. Although not extensively studied in the developing gastric epithelium, its expression in other gastrointestinal contexts implies roles beyond the nervous system. For instance, PGP9.5 may contribute to epithelial–neural crosstalk during organ morphogenesis, and has also been implicated in intracellular protein turnover and stress responses during periods of rapid cellular remodeling, such as tissue repair and differentiation [39,40].

Recent data by Gorecki et al. demonstrated that PGP9.5 marks proliferating neuroblasts in the adult intestinal myenteric plexus and co-localizes with cell cycle markers, suggesting a dual role in neural identity and epithelial-neuronal plasticity [42]. The altered spatial and temporal expression patterns of PGP9.5, particularly the reduced epithelial levels in *yotari* mice at E13.5 and the absence of the typical developmental decline, suggest a potential compensatory or stress-responsive role during early epithelial development in the context of disrupted Reelin–Dab1 signaling. Nevertheless, this interpretation remains hypothetical without direct functional evidence.

Additionally, Oh et al. observed increased PGP9.5 expression in Hirschsprung’s disease, likely indicating compensatory upregulation in response to developmental abnormalities [43]. Similarly, Yamashita et al. linked PGP9.5 promoter methylation to gastric carcinogenesis, reinforcing its role in epithelial maintenance and possibly tumor suppression [44]. Together, these findings suggest that PGP9.5 could act as a stress-response mediator in the developing stomach, although the precise mechanisms and downstream effects are still unclear. Sox2 expression showed no statistically significant differences in either epithelium or mesenchyme between any groups or timepoints, despite semi-quantitative scoring showing increased staining intensity in *yotari* at E15.5 in both compartments. These results suggest that any apparent upregulation is subtle or highly variable, and not robust enough to reach significance.

Sox2 plays a pivotal role in sustaining progenitor cell populations during organogenesis. It regulates symmetric and asymmetric division, influences differentiation trajectories, and promotes regeneration following injury [22,45,46]. In this study, the trend toward increased Sox2 in *yotari* mice may reflect a mild compensatory response, but was not statistically supported. Raghoebir et al. demonstrated Sox2’s capability to redirect intestinal endoderm toward a gastric epithelial fate, highlighting its role in lineage specification [47]. This is particularly relevant in disrupted developmental environments, such as the *yotari* stomach, where maintaining gastric identity may require Sox2 reinforcement to counteract aberrant signals. Sox2 is recognized for its tumor-suppressive functions within the gastric epithelium, where it contributes to the maintenance of epithelial polarity and the prevention of malignant transformation, as demonstrated by Sarkar et al. [46]. Their study showed that Sox2 deletion resulted in increased susceptibility to cellular transformation and disruption of epithelial architecture. While elevated Sox2 expression may play a stabilizing role under conditions of developmental stress, our findings suggest that this function was not significantly engaged at the protein level in *yotari* mice.

The altered expression of Dab1, Reelin, PGP9.5, and Sox2 in *yotari* mice may reflect upstream transcriptional regulation. In pluripotent cells, Sox2 cooperates with Oct4 to regulate key genes during early development [48]. Reelin transcription is activated by Sp1 and Tbr1, with promoter methylation also playing a modulatory role [49]. PGP9.5 (UCHL1) expression is frequently silenced through promoter hypermethylation in epithelial tumors, and can be reactivated by demethylating agents [50,51]. In *yotari* mice, disrupted Reelin–Dab1 signaling may interfere with PI3K/Akt and MAPK pathways, altering transcription factor dynamics and chromatin accessibility, thereby contributing to the compartment-specific expression patterns we observe. However, it is important to note that these observations are correlative and do not establish causation. The proposed “compensatory mechanisms” remain hypothetical without functional validation.

While most existing studies of Reelin–Dab1 signaling during gut development focus on the intestine, recent work has begun to illuminate stomach-specific developmental pathways and ENS integration. Gastric epithelial regionalization, particularly fundus versus antrum specification, is orchestrated by signaling networks including Wnt/β-catenin during embryonic development [52]. Simultaneously, intrinsic and extrinsic components of the ENS establish stomach innervation through sequential colonization by vagal neural crest derivatives and Schwann cell precursors, with coordinated ENS–mesenchyme interactions essential for functional gastrointestinal architecture [53,54]. Although direct connections between Reelin–Dab1 signaling and these stomach-specific developmental processes remain unexplored, acknowledging these advances provides a more comprehensive developmental context for interpreting our findings.

However, certain limitations should be acknowledged. The semi-quantitative assessment of protein expression, although complemented by blinded scoring and inter-observer agreement, remains partly subjective and may not fully capture subtle differences. Moreover, the lack of functional validation experiments, such as rescue assays or pathway inhibition studies, precludes definitive conclusions about the mechanistic roles of Dab1, Reelin, PGP9.5, and Sox2 in gastric development. Finally, no formal tests for normality (e.g., Shapiro–Wilk) or equality of variances (e.g., Levene’s test) were performed prior to ANOVA, which may affect the robustness of the statistical inferences. In addition, the analysis was limited to two developmental stages (E13.5 and E15.5), which, while strategically chosen, do not capture earlier or later developmental events.

## 5. Conclusions

Our findings indicate that Dab1 deficiency leads to distinct molecular alterations in both the epithelial and mesenchymal compartments of the developing stomach, underscoring the critical role of the Reelin–Dab1 signaling axis in coordinating epithelial–mesenchymal interactions during gastric development. Notably, significant differences were observed primarily in the mesenchyme for Dab1 and Reelin and in the epithelium for Dab1 and PGP9.5, suggesting compartment-specific responses to disrupted signaling. The apparent upregulation of PGP9.5 and Sox2 in *yotari* mice may represent possible compensatory responses aimed at maintaining epithelial integrity, though these interpretations remain hypothetical in the absence of functional validation.

Importantly, this study is among the first to examine Dab1 and related protein expression in the developing stomach, filling a significant gap in the literature and providing novel insights into how altered Reelin-Dab1 signaling may impact enteric nervous system formation and gastric tissue organization. However, the absence of functional experiments, such as genetic rescue or pathway inhibition studies, limits our ability to draw definitive conclusions regarding the biological roles of these proteins in development. Future research should prioritize functional assays to determine the mechanistic significance of the observed expression patterns.

## Figures and Tables

**Table 1:** Antibodies used for immunofluorescence.

Antibody Type	Antibody	Host	Dilution	Source
Primary	Anti-Dab1 (ab78200)	Rabbit	1:100	Abcam (Cambridge, UK)
	Anti-Reelin (sc-25346)	Mouse	1:50	Santa Cruz Biotechnology Inc. (Santa Cruz, CA, USA)
	Anti-PGP9.5 (ab8189)	Mouse	1:100	Abcam (Cambridge, UK)
	Anti-Sox2 (3579T)	Rabbit	1:400	Cell Signaling Technology (Danvers, MA, USA)
Secondary	Alexa Fluor^®^ 488 Anti-Rabbit IgG (711-545-152)	Donkey	1:300	Jackson ImmunoResearch Laboratories, Inc. (West Grove, PA, USA)
	Rhodamine Red™-X Anti-Mouse IgG (715-295-151)	Donkey	1:300	Jackson ImmunoResearch Laboratories, Inc. (West Grove, PA, USA)

**Table 2:** Semi-quantitative scoring of Dab1, Reelin, PGP9.5, and Sox2 expression in the embryonic stomach at E13.5 and E15.5.

Antibody	Tissue	E13.5 (ctrl)	E13.5 (*yot*)	E15.5 (ctrl)	E15.5 (*yot*)
Dab1	Epithelium (e)	++	+++	+++	+++
	Mesenchyme (m)	++	+++	+++	+++
Reelin	Epithelium (e)	++	++	++	+
	Mesenchyme (m)	+	+	++	+
PGP9.5	Epithelium (e)	++	+	+	+++
	Mesenchyme (m)	+++	+++	+++	+++
Sox2	Epithelium (e)	++	+	+	+++
	Mesenchyme (m)	++	+	+	+++

+++ strong reactivity; ++ moderate reactivity; + mild reactivity; E—day of embryonic development; ctrl—control specimens; *yot*—*yotari*. Scores represent the average of semi-quantitative evaluations from three embryos per genotype and timepoint (*n* = 3).

## Data Availability

The data supporting the findings of this study are available from the corresponding author upon reasonable request. No publicly archived datasets were used or generated in this study.

## Supplementary Materials

The following supporting information can be downloaded at: https://www.mdpi.com/article/10.3390/genes16091013/s1, Table S1: *Two-way ANOVA results:* Two-way ANOVA analysis of protein expression levels in gastric epithelium and mesenchyme at embryonic stages E13.5 and E15.5 in control and *yotari* mice; Table S2: Tukey’s multiple comparisons: Tukey’s post-hoc multiple comparisons of protein expression levels between developmental stages and genotypes in gastric epithelium and mesenchyme.
