# Supplementary material for: Expression Pattern of Dab1, Reelin, PGP9.5 and Sox2 in the Stomach of Yotari (Dab1−/−) Mice
Source: Genes (Basel). 2025 Aug 27;16(9):1013. doi: 10.3390/genes16091013 (PMC12469321; doi:10.3390/genes16091013)
Supplement: Supplementary file 1 [file genes-16-01013-s001.zip › genes-3800524-supplementary.pdf]

genes

MDPI

.

Supplementary Table S1. ***Two-way ANOVA results:*** Two-way ANOVA analysis of protein expression levels in gastric epithelium and mesenchyme at embryonic stages E13.5 and E15.5 in control and *yotari* mice.

Protein	Compartment	Source of Variation	% of Total Variation	F (DFn, DFd)	p-value	Significance	Mean Difference	95% CI of Difference
**DAB1**	Epithelium	Interaction	6.187	F(1,34) = 8.868	0.0053	**	—	—
		Genotype (*Yot* – Ctrl)	5.457	F(1,34) = 7.822	0.0084	**	+2.218	+1.745 to +2.691
		Stage (E15.5 – E13.5)	63.36	F(1,34) = 90.82	<0.0001	****	+0.651	+0.178 to +1.124
	Mesenchyme	Interaction	0.967	F(1,34) = 0.8893	0.3523	ns	—	—
		Genotype (*Yot* – Ctrl)	20.23	F(1,34) = 18.61	0.0001	***	+1.769	+1.216 to +2.321
		Stage (E15.5 – E13.5)	46.04	F(1,34) = 42.34	<0.0001	****	+1.173	+0.620 to +1.725
**REELIN**	Epithelium	Interaction	1.423	F(1,34) = 0.5974	0.4449	ns	—	—
		Genotype (*Yot* – Ctrl)	15.31	F(1,34) = 6.429	0.0160	*	-0.697	-2.278 to +0.885
		Stage (E15.5 – E13.5)	1.907	F(1,34) = 0.8009	0.3771	ns	+1.974	+0.392 to +3.555
	Mesenchyme	Interaction	1.003	F(1,34) = 1.288	0.2643	ns	—	—
		Genotype (*Yot* – Ctrl)	21.41	F(1,34) = 27.50	<0.0001	****	-9.131	-11.51 to -6.752

**Protein**	**Compartment**	**Source of Variation**	**% of Total Variation**	**F (DFn, DFd)**	**p-value**	**Significance**	**Mean Difference**	**95% CI of Difference**
		Stage (E15.5 – E13.5)	47.35	F(1,34) = 60.82	<0.0001	****	+6.140	+3.760 to +8.519
**PGP9.5**	Epithelium	Interaction	10.99	F(1,36) = 6.696	0.0138	*	—	—
		Genotype (*Yot* – Ctrl)	13.86	F(1,36) = 8.442	0.0062	**	-4.445	-7.329 to -1.562
		Stage (E15.5 – E13.5)	16.05	F(1,36) = 9.776	0.0035	**	-4.131	-7.014 to -1.247
	Mesenchyme	Interaction	15.74	F(1,36) = 9.026	0.0048	**	—	—
		Genotype (*Yot* – Ctrl)	1.586	F(1,36) = 0.910	0.3466	ns	-4.155	-6.649 to -1.660
		Stage (E15.5 – E13.5)	19.89	F(1,36) = 11.41	0.0018	**	-1.173	-3.668 to +1.322
**SOX2**	Epithelium	Interaction	4.502	F(1,36) = 1.722	0.1978	ns	—	—
		Genotype (*Yot* – Ctrl)	0.0253	F(1,36) = 0.0097	0.9222	ns	+0.364	-0.669 to +1.396
		Stage (E15.5 – E13.5)	1.334	F(1,36) = 0.5101	0.4797	ns	-0.050	-1.082 to +0.982
	Mesenchyme	Interaction	0.0017	F(1,36) = 0.0007	0.9787	ns	—	—
		Genotype (*Yot* – Ctrl)	10.15	F(1,36) = 4.211	0.0475	*	+0.594	-0.474 to +1.662
		Stage (E15.5 – E13.5)	3.064	F(1,36) = 1.271	0.2670	ns	-1.081	-2.149 to -0.013

Two-way ANOVA was applied to determine the effects of genotype (control vs. *yotari*), developmental stage (E13.5 vs. E15.5), and their interaction on the expression of the investigated proteins (Dab1, Reelin, PGP9.5,

and Sox2) in both epithelial and mesenchymal compartments of the developing stomach. The table reports F values, degrees of freedom, and p-values for each factor and their interaction. A significant genotype effect indicates differences between control and *yotari* groups across both stages; a significant stage effect reflects developmental changes between E13.5 and E15.5 independent of genotype; and a significant interaction indicates that the effect of genotype depends on developmental stage. Significant results ($p < 0.05$) are highlighted.

Supplementary Table S2. Tukey's multiple comparisons: Tukey's post-hoc multiple comparisons of protein expression levels between developmental stages and genotypes in gastric epithelium and mesenchyme.

Protein	Compartment	Comparison	Mean Difference	95% CI of Difference	Adjusted p-value	Significance
**Dab1**	Epithelium	E13.5 Ctrl vs. E13.5 *Yot*	-1.525	-2.440 to -0.6101	0.0004	***
		E13.5 Ctrl vs. E15.5 Ctrl	0.04216	-0.8726 to 0.9569	0.9993	ns
		E13.5 *Yot* vs. E15.5 *Yot*	-1.344	-2.206 to -0.4816	0.0010	***
		E15.5 Ctrl vs. E15.5 *Yot*	-2.911	-3.773 to -2.049	<0.0001	****
**Dab1**	Mesenchyme	E13.5 Ctrl vs. E13.5 YOT	-2.025	-3.094 to -0.9568	<0.0001	****
		E13.5 Ctrl vs. E15.5 Ctrl	-1.429	-2.497 to -0.3605	0.0051	**
		E13.5 *Yot* vs. E15.5 *Yot*	-0.9162	-1.923 to 0.09105	0.0855	ns
		E15.5 Ctrl vs. E15.5 *Yot*	-1.512	-2.520 to -0.5052	0.0015	**
**Reelin**	Epithelium	E13.5 Ctrl vs. E13.5 *Yot*	0.09499	-2.964 to 3.154	0.9998	ns

**Protein**	**Compartment**	**Comparison**	**Mean Difference**	**95% CI of Difference**	**Adjusted p-value**	**Significance**
		E13.5 Ctrl vs. E15.5 Ctrl	-2.575	-5.634 to 0.4840	0.1244	ns
		E13.5 *Yot* vs. E15.5 *Yot*	-1.372	-4.256 to 1.512	0.5787	ns
		E15.5 Ctrl vs. E15.5 *Yot*	1.298	-1.586 to 4.182	0.6214	ns
**Reelin**	Mesenchyme	E13.5 Ctrl vs. E13.5 *Yot*	10.46	5.858 to 15.06	<0.0001	****
		E13.5 Ctrl vs. E15.5 CTRL	-4.811	-9.413 to -0.2092	0.0377	*
		E13.5 YOT vs. E15.5 *Yot*	-7.469	-11.81 to -3.130	0.0003	***
		E15.5 Ctrl vs. E15.5 *Yot*	7.802	3.464 to 12.14	0.0001	***
**PGP9.5**	Epithelium	E13.5 Ctrl vs. E13.5 *Yot*	8.125	2.709 to 13.54	0.0015	**
		E13.5 Ctrl vs. E15.5 Ctrl	7.810	2.395 to 13.23	0.0023	**
		E13.5 YOT vs. E15.5 *Yot*	0.4517	-4.963 to 5.867	0.9959	ns
		E15.5 Ctrl vs. E15.5 *Yot*	0.7662	-4.649 to 6.181	0.9809	ns
**PGP9.5**	Mesenchyme	E13.5 Ctrl vs. E13.5 *Yot*	7.850	3.165 to 12.54	0.0004	***
		E13.5 Ctrl vs. E15.5 Ctrl	4.869	0.1837 to 9.554	0.0391	*
		E13.5 *Yot* vs. E15.5 *Yot*	-2.522	-7.208 to 2.163	0.4775	ns

**Protein**	**Compartment**	**Comparison**	**Mean Difference**	**95% CI of Difference**	**Adjusted p-value**	**Significance**
		E15.5 Ctrl vs. E15.5 *Yot*	0.4591	-4.226 to 5.144	0.9934	ns
**Sox2**	Epithelium	E13.5 Ctrl vs. E13.5 *Yot*	0.3043	-1.634 to 2.243	0.9742	ns
		E13.5 Ctrl vs. E15.5 Ctrl	0.7179	-1.221 to 2.657	0.7518	ns
		E13.5 YOT vs. E15.5 *Yot*	-0.6178	-2.556 to 1.321	0.8261	ns
		E15.5 Ctrl vs. E15.5 *Yot*	-1.031	-2.970 to 0.9072	0.4877	ns
**Sox2**	Mesenchyme	E13.5 Ctrl vs. E13.5 YOT	-0.6080	-2.614 to 1.398	0.8464	ns
		E13.5 Ctrl vs. E15.5 CTRL	1.067	-0.9395 to 3.073	0.4883	ns
		E13.5 YOT vs. E15.5 *Yot*	1.095	-0.9111 to 3.101	0.4656	ns
		E15.5 Ctrl vs. E15.5 *Yot*	-0.5796	-2.586 to 1.427	0.8638	ns

Following two-way ANOVA, Tukey's post-hoc test was performed to assess pairwise differences between groups. Comparisons were made between control and *yotari* mice at the same developmental stage, as well as between E13.5 and E15.5 stages within each genotype, for all analyzed proteins (Dab1, Reelin, PGP9.5, and Sox2) in both epithelial and mesenchymal compartments. The table provides mean differences, 95% confidence intervals (CI), standard error of the difference, adjusted p-values, and q statistics. Significance levels are indicated as: ns = not significant, * $p < 0.05$, ** $p < 0.01$, *** $p < 0.001$, **** $p < 0.0001$. These results specify which group differences drive the main effects and interactions detected in the two-way ANOVA.
